# Practical approach to primary retroperitoneal masses in adults

**DOI:** 10.1590/0100-3984.2017.0179

**Published:** 2018

**Authors:** Micaela Maciel dos Santos Mota, Regis Otaviano França Bezerra, Marcio Ricardo Taveira Garcia

**Affiliations:** 1 Serviço de Radiologia do Instituto do Câncer do Estado de São Paulo Octavio Frias de Oliveira (Icesp), São Paulo, SP, Brazil.

**Keywords:** Retroperitoneal space/anatomy & histology, Retroperitoneal neoplasms/diagnosis, Tomography, X-ray computed, Magnetic resonance imaging

## Abstract

Primary retroperitoneal masses constitute a heterogeneous group of uncommon lesions and represent a challenge due to overlapping imaging findings. Most are malignant lesions. Although they are more prevalent in adults, they can occur at any age. Such lesions are classified as primary when they do not originate from a specific retroperitoneal organ and are divided, according to the image findings, into two major groups: solid and cystic. The clinical findings are nonspecific and vary depending on the location of the lesion in relation to adjacent structures, as well as on its behavior. The main imaging methods used for staging and surgical planning, as well as for selecting the biopsy site and guiding the biopsy procedure, are computed tomography and magnetic resonance imaging. In most cases, the treatment is challenging, because of the size of the lesions, vascular involvement, or involvement of adjacent organs. In this article, we present a review of the retroperitoneal anatomy and a practical approach to the main imaging features to be evaluated, with a view to the differential diagnosis, which can guide the clinical management.

## INTRODUCTION

Retroperitoneal masses constitute a heterogeneous group of lesions, originating in the retroperitoneal spaces, that pose a diagnostic challenge for radiologists^(^^1^^)^. The majority of cases are malignant tumors, of which approximately 75% are mesenchymal in origin^(^^2^^-^^4^^)^. Although such tumors are more prevalent in adults, they can occur at any age^(^^2^^)^.

When they do not originate from organs such as the kidneys, adrenal glands, pancreas, or bowel loops, retroperitoneal masses are classified as primary and are categorized as solid or cystic (Table 1), depending on their appearance on imaging^(^^5^^,^^6^^)^. Solid lesions can be divided into four groups, by origin^(^^3^^)^: mesenchymal, neural, germ-cell, and lymphoproliferative. Among the cystic lesions, the most common are lymphangioma and cystic mesothelioma^(^^3^^,^^7^^,^^8^^)^. There are also non-neoplastic processes, primarily retroperitoneal fibrosis, non-Langerhans histiocytosis (Erdheim-Chester disease), and extramedullary hematopoiesis.

**Table 1:** Categories and subcategories of predominantly solid and predominantly cystic retroperitoneal lesions.

Category	Retroperitoneal lesions in adults	
	Solid	Cystic
Neoplastic	Lymphoma	Lymphangioma
	Liposarcoma	Cystadenoma
	Malignant fibrous histiocytoma	Cystadenocarcinoma
	Leiomyosarcoma	Cystic mesothelioma
	Neurogenic tumors	Mature teratoma
	Germ-cell tumors	
Non neoplastic	Retroperitoneal fibrosis	Epidermoid cyst
	Extramedullary hematopoiesis	Epidermoid cyst
	Erdheim-Chester disease	Non-pancreatic pseudocyst
		Bronchogenic cyst
		Lymphoceles, urinomas, hematomas

The clinical manifestations of retroperitoneal masses are nonspecific, depending on their location and relation with the adjacent structures^(^^9^^)^. The main imaging methods for the evaluation of these lesions are computed tomography (CT) and magnetic resonance imaging (MRI), imaging features facilitating the differential diagnosis, the tumor staging, and the definition of the surgical strategy, as well as guiding biopsies^(^^1^^,^^5^^,^^10^^,^^11^^)^. Although there is significant overlapping of the imaging findings and the final diagnosis is defined by histopathological analysis, there are characteristics that are specific to certain lesions and can guide clinical practice. The treatment of retroperitoneal masses is challenging, mainly due to their anatomical location, dimensions, vascular involvement, or involvement of adjacent organs^(^^12^^,^^13^^)^.

In this article, we present a review of the retroperitoneal anatomy. We also take a practical approach to the imaging characteristics to be evaluated in, as well as the clinical management of, the main primary retroperitoneal masses in adults.

## RETROPERITONEAL ANATOMY

**Figure 1:**
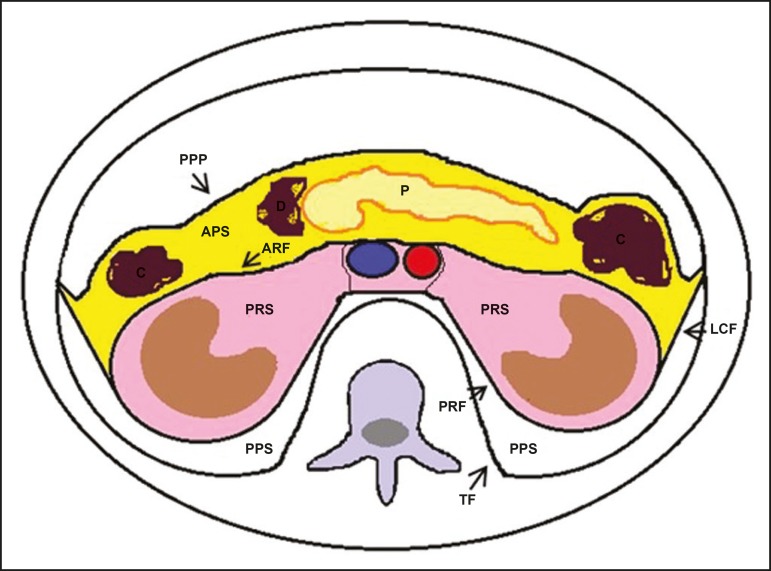
Retroperitoneal anatomy. The *anterior pararenal space* is delimited anteriorly by the posterior parietal peritoneum, posteriorly by the anterior renal fascia, and laterally by the lateroconal fascia. It includes the pancreas (P) and second portion of the duodenum (D), as well as the ascending and descending colon (C). The *posterior pararenal space* is delimited anteriorly by the posterior renal fascia and posteriorly by the transversal fascia. It contains fat. The *perirenal space*, which contains the kidneys and adrenal glands, is located between the anterior and posterior renal fasciae. The *central region* includes the aorta and inferior vena cava, as well as lymphatic chains and nerve structures. APS, anterior pararenal space; PRS, perirenal space; PPS, posterior pararenal space; PPP, posterior parietal peritoneum; ARF, anterior renal fascia; PRF, posterior renal fascia; LCF, lateroconal fascia; TF, transverse fascia.

The retroperitoneal space extends from the diaphragm to the pelvis, being delimited posteriorly by the transverse fascia and anteriorly by the posterior parietal peritoneum, and is divided into compartments^(^^11^^,^^14^^,^^15^^)^, as depicted in Figure 1. The anterior pararenal space, which is delimited anteriorly by the posterior parietal peritoneum, posteriorly by the anterior renal fascia, and laterally by the lateroconal fascia, includes the pancreas and the second portion of the duodenum, as well as the ascending and descending colon. The posterior pararenal space, which is delimited anteriorly by the posterior renal fascia and posteriorly by the transverse fascia, contains fat. The perirenal space, which is located between the anterior and posterior renal fasciae, contains the kidneys and adrenal glands. The retroperitoneum also includes a central region, which contains the aorta and inferior vena cava, as well as lymphatic chains and the nerve plexuses.

## RADIOLOGICAL EVALUATION OF RETROPERITONEAL MASSES

In the initial evaluation of a retroperitoneal mass, its location within the retroperitoneal space should be confirmed and the affected compartment (e.g., the anterior pararenal space) should be identified^(^^3^^)^. Findings of anterior displacement of abdominal structures, such as the aorta or colon, or retroperitoneal organs, such as the kidneys, help identify the lesion site. However, there are situations in which it is difficult to determine the exact location, because of the anatomical distortion caused by the lesion^(^^1^^,^^3^^,^^5^^,^^16^^)^. In such cases, retroperitoneal involvement should be detailed by describing the spaces involved.

To categorize a retroperitoneal mass as a primary retroperitoneal lesion, its origin from a larger retroperitoneal organ should be excluded. It should then be classified as solid or cystic, its main imaging characteristics (macroscopic fat, calcifications, myxoid stroma, necrosis, and cystic areas of vascularization) should be evaluated, and its relationship with adjacent structures should be described. There are radiological signs (the crescent sign, embedded organ sign, and phantom organ sign) that aid in the diagnostic assessment; the absence of those signs can confirm the categorization of a mass as a primary retroperitoneal lesion^(^^1^^)^.The collective evaluation of these findings is aimed at narrowing the possible differential diagnoses and guiding the therapeutic planning^(^^1^^,^^3^^,^^5^^,^^14^^)^.

### Fat

A finding of intralesional fat significantly shortens the list of differential diagnoses, narrowing it down to only lesions with distinct biological behavior, such as liposarcoma, teratoma, and extramedullary hematopoiesis^(^^17^^)^.

Liposarcoma - Liposarcoma is the most common retroperitoneal sarcoma, accounting for approximately 30% of all retroperitoneal sarcomas. It affects individuals in the fifth and sixth decades of life. It can be classified as well differentiated, with or without dedifferentiated, myxoid, round cell, or pleomorphic components, which have distinct clinical and radiological characteristics. It is often located in the perirenal space.

Well-differentiated liposarcoma, which is the most common subtype of liposarcoma, contains mature adipose tissue and is characterized by infiltration of the adjacent structures^(^^3^^,^^4^^,^^14^^,^^18^^)^. Among the imaging characteristics that favor the diagnosis, making a benign lesion less likely, is lesion size greater than 10 cm, the presence of thick (> 0.2 cm) septa, and foci of nodular enhancement^(^^3^^,^^19^^,^^20^^)^. However, histopathological analysis with the molecular markers (anti-CDK4 and anti-MDM2 antibodies) facilitates that distinction. When technically feasible, the treatment of choice is surgical resection, with wide negative margins to avoid local recurrence^(^^21^^,^^22^^)^. Currently, some therapies targeting amplified oncogenes have shown promise in the treatment of certain liposarcomas, especially the well-differentiated and dedifferentiated subtypes^(^^23^^,^^24^^)^.

**Figure 2:**
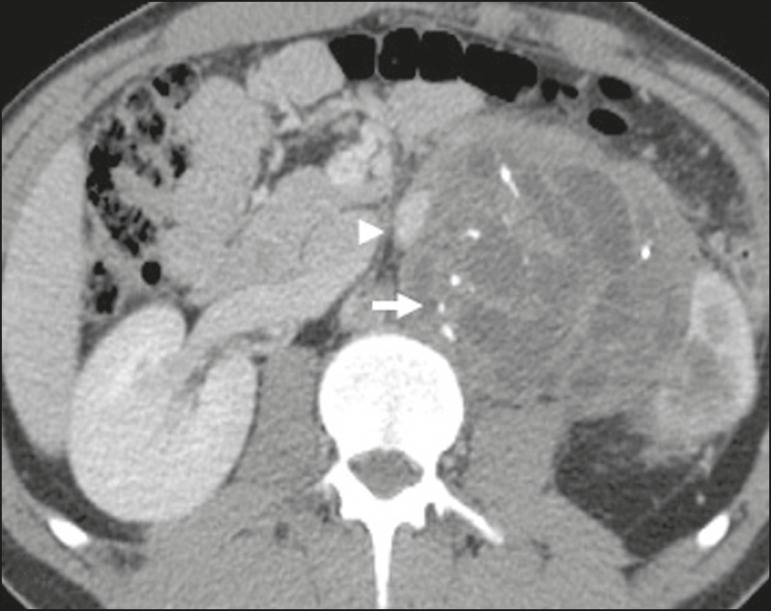
Mature teratoma in a 23-year-old female. CT scan showing a retroperitoneal mass with fat components, cystic areas, and calcifications (arrow). These findings, especially the fat component, are suggestive of a germ-cell origin. Note the anterior displacement of the aorta in relation to the vertebral body-an indirect sign of retroperitoneal location (arrowhead). The diagnosis was confirmed by percutaneous biopsy.

**Retroperitoneal teratoma** - Retroperitoneal teratoma is a germ-cell tumor, derived from the embryonic layers, than can present elevated serum levels of markers, including alpha-fetoprotein, CEA, CA-19-9, and β-hCG^(^^6^^,^^25^^,^^26^^)^. It is characterized by macroscopic fat, cystic areas, calcifications, and a fat-fluid level, as well as heterogeneous contrast enhancement^(^^26^^,^^27^^)^, as shown in Figure 2. Surgical excision of the tumor is the main treatment^(^^6^^,^^25^^)^. In male patients, consideration should be given to the possibility of secondary retroperitoneal lesion of gonadal origin and the testes should always be investigated^(^^5^^)^.

**Extramedullary hematopoiesis** - Extramedullary hematopoiesis is a compensatory mechanism related to reduced hematopoiesis in the bone marrow and is characterized by deposits of hematopoietic tissue in organs of mesenchymal origin (the spleen and liver) and within paravertebral tissues^(^^16^^)^. Imaging can reveal multiple, homogeneous, bilateral masses, with minimal contrast enhancement, with or without macroscopic fat^(^^5^^)^. Secondary findings of chronic anemia, including clinical and laboratory data, contribute to the diagnosis.

**Extra-adrenal myelolipoma** - Extra-adrenal myelolipoma is a rare, typically unilateral, benign mesenchymal lesion, containing adipose and hematopoietic tissue, that can occur in the retroperitoneum, most commonly in the presacral region. It usually affects women between 50 and 70 years of age. Most patients with extra-adrenal myelolipoma are asymptomatic, the lesion usually being identified as an incidental finding on an imaging examination, although some patients present with abdominal pain, due to hemorrhage, tumor infarction, or extrinsic compression. The main differential diagnoses are retroperitoneal teratoma, extramedullary hematopoiesis, and liposarcoma, the last being practically indistinguishable from extra-adrenal myelolipoma on imaging studies. Biopsy is often required in order to make that distinction, and the presence of hematopoietic components confirms the diagnosis of extra-adrenal myelolipoma. Treatment options range from imaging follow-up-for small, asymptomatic lesions-to surgery-for symptomatic, bulky, or progressively growing lesions, as well as for cases in which the nature of the lesion remains undetermined^(^^21^^,^^28^^-^^30^^)^.

**Cystic lymphangioma** - On imaging studies, cystic lymphangioma can show lipid content, due to the presence of chylous ascites^(^^8^^)^.

**Key point:** There is a specific group of retroperitoneal lesions that contain fat and have distinct biological behavior. The identification of fat content during the imaging evaluation reduces the number of possible differential diagnoses.

### Calcifications

Among the solid lesions that can contain calcifications are neurogenic tumors, dedifferentiated liposarcomas, teratoma, and undifferentiated pleomorphic sarcoma^(^^5^^,^^18^^)^.

**Figure 3:**
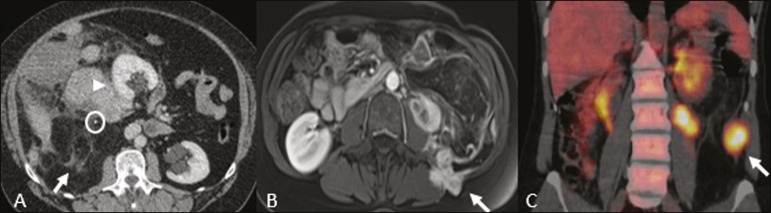
Dedifferentiated liposarcoma. **A:** 58-year-old male. CT scan showing an infiltrative lesion with solid and fat components (arrow).Note the displacement of the right kidney (arrowhead), inferring location in the posterior pararenal space, and the focus of calcification (circle) suggesting dedifferentiation of the tumor. **B,C:** 44-year-old female. Contrast-enhanced T1-weighted MRI sequence with fat saturation (**B**) showing a fat-containing infiltrative lesion (signal loss) and nodules infiltrating the abdominal wall (arrow). An ultrasound-guided percutaneous biopsy was performed, and an ^18^F-FDG PET/CT scan showed hypermetabolism (arrow in **C**), corresponding to the foci of dedifferentiation.

**Dedifferentiated liposarcoma** - Dedifferentiated liposarcoma presents calcifications in approximately 30% of cases; when accompanied by nodules with heterogeneous enhancement (Figure 3), such calcifications are suggestive of areas of tumor dedifferentiation and of a worse prognosis^(^^2^^,^^18^^,^^31^^,^^32^^)^. In the investigation of liposarcomas, ^18^F-FDG PET/CT can facilitate the evaluation of the tumor grade, as well as the staging and postoperative follow-up, through quantification of the FDG uptake of the solid components of the lesion, especially in the dedifferentiated and pleomorphic subtypes^(^^18^^,^^22^^,^^32^^)^. Some authors have suggested that the tumor grade correlates with the rate of glycolysis^(^^22^^)^.

**Retroperitoneal teratoma** - Retroperitoneal teratoma is characterized by a lesion that is predominantly cystic, containing fat and calcifications, a finding that is suggestive of mature teratoma (Figure 2). In contrast, immature teratomas are uncommon and cannot be distinguished from the benign variant on the basis of the imaging characteristics alone^(^^3^^,^^5^^,^^33^^)^.

**Extragastrointestinal stromal tumor** - An extragastrointestinal stromal tumor is a mesenchymal neoplasm originating from the interstitial cells of Cajal, typically located outside the gastrointestinal tract and rarely involving the retroperitoneum. It is characterized by mutations in the c-kit gene, being histologically similar to a gastrointestinal stromal tumor. Extragastrointestinal stromal tumors manifest as homogeneous or heterogeneous masses, with central necrosis and calcifications. The main differential diagnoses include sarcomas^(^^18^^)^.

**Key point:** Calcification is a common finding in most retroperitoneal lesions and, when accompanied by other imaging features, such as fat, narrows the differential diagnosis. Regarding liposarcoma, the presence of calcification, nodules with enhancement and FDG uptake in the ^18^F-FDG PET/CT study suggest a dedifferentiated tumor component.

### Myxoid stroma

A myxoid stroma consists of a mucopolysaccharide-rich matrix and is characterized by a hyperintense signal in T2-weighted MRI sequences and by delayed contrast enhancement^(^^1^^)^. The major lesions that contain a myxoid matrix are neurogenic tumors, myxoid liposarcoma, and myxofibrosarcoma^(^^2^^,^^31^^)^. We highlight myxoid liposarcoma and neurogenic tumors, which can originate from the neural sheath, the paraganglion system, or the sympathetic ganglia^(^^3^^)^.

**Neurogenic tumors** - Neurogenic tumors originate from the neural sheath, encompassing schwannomas and neurofibromas.

Schwannomas are the most common benign neural sheath tumors, are common in young adults, and can be accompanied by neurofibromatosis^(^^1^^,^^10^^,^^11^^,^^34^^)^. On imaging examinations, schwannomas manifest as circumscribed masses with oval or spherical morphology and heterogeneous enhancement, commonly located in the paravertebral region^(^^3^^,^^11^^)^. On MRI, the signal intensity varies depending on the predominant tissue type; for example, the characteristic hyperintense signal in T2-weighted sequences is related to a greater quantity of myxoid stroma^(^^1^^,^^10^^)^. Depending on their location and size, schwannomas can also compress adjacent structures ^(^^10^^,^^34^^)^. The treatment of choice is surgery-mainly for lesions that are large or symptomatic-or monitoring-for asymptomatic cases or cases with a high risk of surgical morbidity^(^^3^^,^^4^^,^^13^^)^.

Neurofibromas are characterized by proliferation throughout all elements of the nerve structure and tend to cause diffuse nerve enlargement^(^^10^^)^. On MRI, they manifest as masses with soft-tissue attenuation, producing hypointense signals on T1-weighted images and signals of variable intensity on T2-weighted images^(^^3^^)^. Other features of neurofibromas include the presence of myxoid degeneration, the target sign, and contrast enhancement^(^^3^^,^^27^^)^. They usually present spindle cell morphology with longitudinal orientation in relation to the affected nerve trajectory, or enlargement of the neural foramen when they originate from the spinal nerve roots^(^^3^^)^. Retroperitoneal plexiform neurofibromas are typically bilateral, symmetrical lesions that follow the lumbosacral plexus distribution, being caused almost exclusively by neurofibromatosis type I^(^^3^^,^^11^^)^. It is of note that malignant peripheral nerve sheath tumors account for 10% of soft-tissue sarcomas and currently represent a group of neoplasms originating from any cell in the neural sheath, including malignant schwannoma and neurofibrosarcomas. Such tumors affect individuals between 20 and 50 years of age and are strongly associated with neurofibromatosis type I^(^^35^^,^^36^^)^.

**Figure 4:**
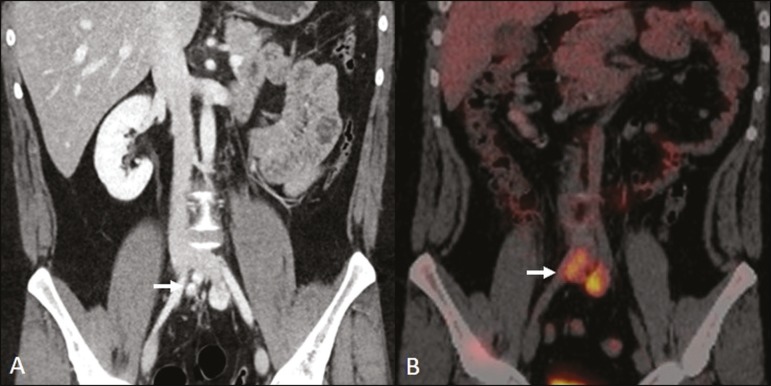
Retroperitoneal paraganglioma in a 24-year-old male. CT and ^18^F-FDG PET/CT scans (**A** and **B**, respectively) showing hypervascular nodular lesions with hyperglycolysis adjacent to the aortoiliac bifurcation.

Among the neurogenic tumors originating from the paraganglion system, we highlight retroperitoneal paragangliomas, which originate from chromaffin cells remaining at extra-adrenal sites. The retroperitoneum is the most common extra-adrenal location of paragangliomas, most commonly arising from the organ of Zuckerkandl, which is located in the para-aortic region near the origin of the inferior mesenteric artery^(^^3^^,^^11^^,^^14^^)^. They are characterized by heterogeneous masses, with hemorrhage, central necrosis (in 40% of cases), punctate calcifications, and a fluid-fluid level. On MRI, they present hyperintense or heterogeneous signals in T2-weighted sequences and intense contrast enhancement^(^^3^^,^^5^^,^^11^^)^. In some cases, there is catecholamine hypersecretion^(^^2^^,^^3^^,^^33^^)^. Approximately 40% of retroperitoneal paragangliomas are malignant, usually leading to necrosis and distant metastases^(^^27^^,^^33^^,^^37^^)^. Functional imaging studies are useful for localization of the tumor and of distant metastases, the methods including MIBG scintigraphy, ^18^F-FDG PET/CT (Figure 4), and ^68^Ga-DOTATATE PET/CT, the last being superior for lesion detection^(^^38^^,^^39^^)^. Surgical resection is the treatment of choice, even in cases with metastasis, because it is associated with improved survival^(^^3^^,^^37^^)^.

**Figure 5:**
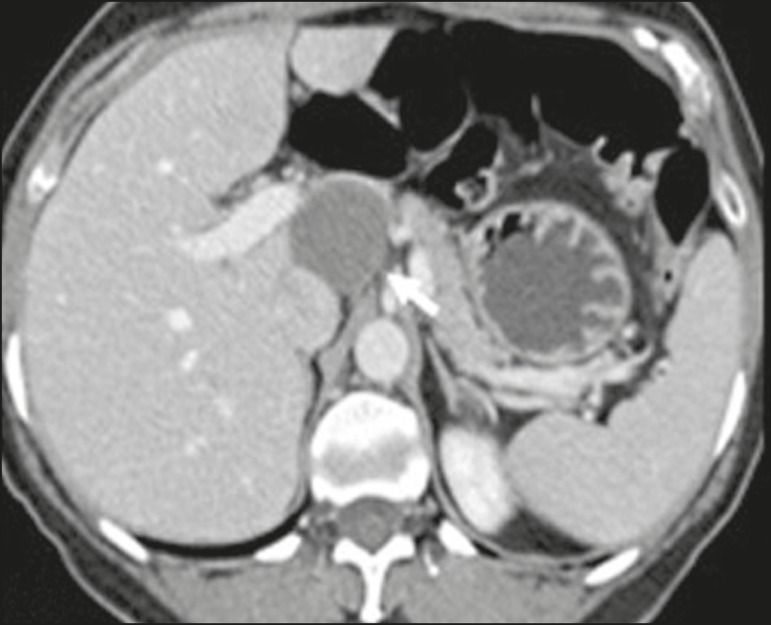
Ganglioneuroma in a 31-year-old female. CT scan showing a retroperitoneal lesion with a pseudocystic aspect (arrow).

The group of neurogenic tumors derived from sympathetic ganglia comprises ganglioneuromas, neuroblastomas, and ganglioneuroblastomas, the last two being more common in pediatric patients^(^^27^^)^. Ganglioneuromas are benign, slow-growing tumors located along the paravertebral sympathetic plexus, usually in the posterior mediastinum or retroperitoneum. A ganglioneuroma manifests as a well-defined mass, with lower attenuation than that of muscle, containing myxoid stroma and punctate calcifications^(^^1^^,^^3^^)^, as depicted in Figure 5. On MRI, ganglioneuromas show a homogeneous, hypointense signal in T1-weighted sequences and a variable signal in T2-weighted sequences. In half of all cases, they are functional, producing catecholamines and androgens^(^^2^^,^^11^^)^. The diagnosis is based on the results of the pathology study, and complete surgical resection is the treatment of choice, especially when the patient is symptomatic^(^^40^^)^. However, there are specialists who advocate for follow-up imaging evaluations, which should be done with caution because of the possibility that there are also poorly differentiated components^(^^40^^,^^41^^)^.

**Myxoid liposarcoma** - Myxoid liposarcoma affects individuals in the 40- to 60-year age group and is more common in the extremities, especially in the deep soft tissues of the thigh, although it can occasionally arise in the retroperitoneum^(^^11^^,^^31^^)^. It contains adipocytic and non-adipocytic myxoid components, which create a pseudocystic appearance on imaging studies, as well as showing slow, progressive contrast enhancement^(^^3^^,^^11^^,^^18^^)^. The adipose content usually corresponds to less than 10% of the tumor volume^(^^31^^)^.

**Myxofibrosarcoma** - Formerly known as myxoid malignant fibrous histiocytoma, myxofibrosarcoma affects individuals with mean age of 65 years, originating in the retroperitoneum in approximately 8% of cases. The imaging findings are nonspecific, with a variable pattern due to the presence of myxoid stroma, fibrous tissue, cellular tissue, hemorrhage, and necrosis, with or without a fluid-fluid level or pseudocapsule^(^^31^^)^.

**Key point:** Myxoid stroma is a common finding in a large group of retroperitoneal tumors and is characterized by the pseudocystic aspect and the characteristic hyperintense signal in T2-weighted MRI sequences. The anatomical relationships of the lesion, such as with nerve structures or with the organ of Zuckerkandl, help narrow the differential diagnosis, as does the presence of genetic syndromes.

### Necrosis

Necrosis is a characteristic finding of high-grade neoplasms, generally indicating a poor prognosis and occurring in the most common malignant retroperitoneal tumors^(^^1^^,^^3^^)^. Such tumors are described below.

**Figure 6:**
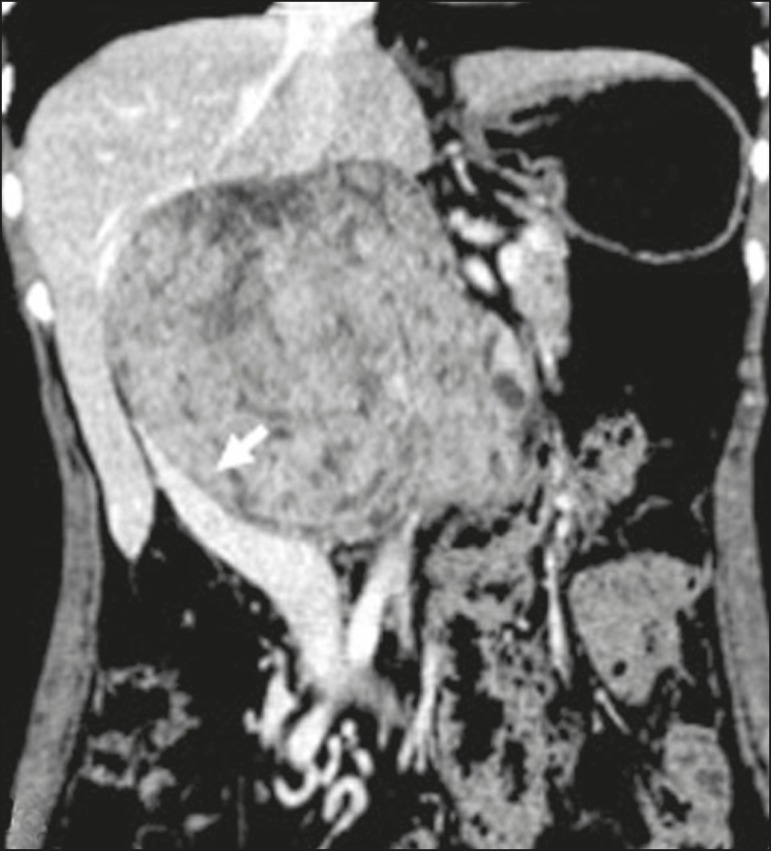
Retroperitoneal leiomyosarcoma in a 56-year-old female. CT scan showing a bulky retroperitoneal lesion with heterogeneous enhancement and foci of necrosis. Note the extensive contact with the inferior vena cava (arrow).

**Leiomyosarcoma** - Leiomyosarcoma is the second most common primary retroperitoneal tumor in adults between 50 and 60 years of age and originates from retroperitoneal smooth muscle tissue, from the vascular wall, or from embryonic remnants of the Wolffian ducts^(^^11^^,^^14^^,^^18^^)^. On imaging, it is characterized by a bulky mass with necrosis, with or without hemorrhage, and contiguous involvement of the vascular structure (Figure 6), calcifications and intralesional fat being uncommon^(^^3^^,^^18^^,^^32^^)^. On imaging studies, three vascular growth patterns have been described^(^^11^^,^^19^^)^: extraluminal, intraluminal, and mixed. Although surgical treatment is complex, given the difficulty related to the size and extent of the tumor, surgery remains the treatment of choice. Tumor recurrence is common^(^^19^^,^^42^^)^. Chemotherapy is also a therapeutic option, even if it is only palliative^(^^13^^,^^22^^,^^43^^)^.

**Undifferentiated pleomorphic sarcoma** - The disease formerly known as malignant fibrous histiocytoma was reclassified as undifferentiated pleomorphic sarcoma by the World Health Organization in 2013^(^^31^^)^. Undifferentiated pleomorphic sarcoma is considered the third most common retroperitoneal tumor and usually affects individuals between 50 and 70 years of age^(^^11^^,^^31^^)^. The imaging findings are characterized by bulky, infiltrative masses with heterogeneous enhancement and areas of necrosis, hemorrhage, fibrosis, and calcifications (in 20% of cases), with or without a pseudocapsule^(^^4^^,^^5^^,^^18^^,^^31^^)^. The treatment of choice is surgical resection, and the prognosis is directly related to tumor grade, lesion size, and metastatic status^(^^19^^)^.

**Pleomorphic liposarcoma** - Pleomorphic liposarcoma is an aggressive, heterogeneous tumor that is the least common liposarcoma subtype, characterized on imaging by areas of necrosis and the absence of adipose components, making it indistinguishable from other solid retroperitoneal tumors^(^^3^^,^^14^^,^^18^^)^.

**Key point:** Liposarcoma, leiomyosarcoma, and undifferentiated pleomorphic sarcoma are the most common malignant mesenchymal tumors of the retroperitoneum, necrosis being a characteristic common to this group and denoting aggression.

### Cystic aspect

Some retroperitoneal tumors have a cystic appearance. In the differential diagnosis of such tumors, lymphangioma is the main lesion to be considered.

**Figure 7:**
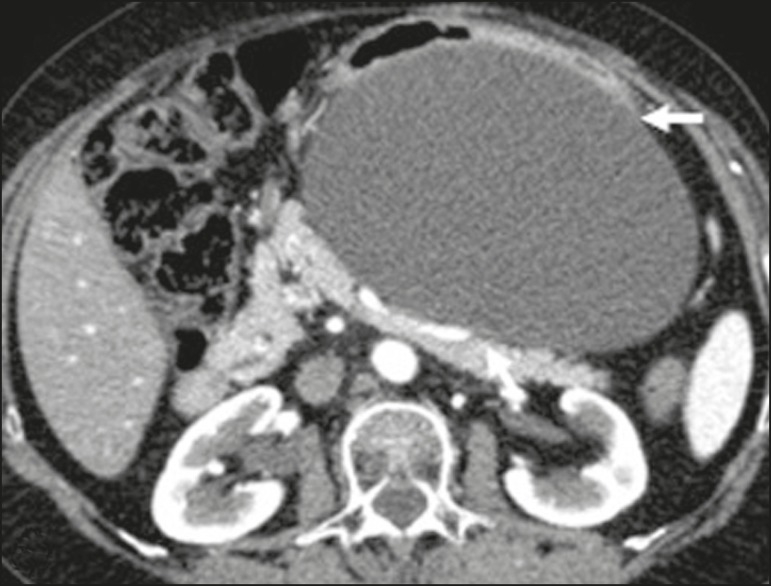
Retroperitoneal cystic lymphangioma in a 37-year-old male. CT scan showing a retroperitoneal cystic lesion (arrow), displacing the bowel loops anteriorly and in contact with the pancreas. Surgical resection was performed, and the pathology showed proliferation of dilated lymphatic vessels, without atypia, consistent with lymphangioma.

**Cystic lymphangioma** - Cystic lymphangioma is a benign, slow-growing lymphatic malformation caused by failure of the communication between the retroperitoneal lymphatic channels and the main lymphatic system^(^^33^^)^. It can present serous, chylous, hemorrhagic, or mixed contents and is characterized by a thin-walled, unilocular or multilocular cystic mass, with variable attenuation depending on its content (Figure 7). In patients who have bulky, rapidly growing lesions or are symptomatic, the treatment of choice is surgical resection. For other patients, conservative management and imaging follow-up should be considered^(^^3^^,^^4^^,^^8^^)^.

**Cystic mesothelioma** - Cystic mesothelioma is a rare benign neoplasm of mesothelial origin and is characterized by thin-walled cystic lesions of varying dimensions that are indistinguishable from other cystic masses of the retroperitoneum^(^^14^^,^^33^^)^. Although cases of malignant transformation have been reported, some authors argue that cystic mesothelioma has no malignant potential^(^^44^^)^. Therefore, periodic imaging follow-up is recommended for patients who are not candidates for surgery. Other less common cystic lesions include Müllerian cysts, epidermoid cysts, nonpancreatic pseudocysts, cystadenoma, and cystadenocarcinoma^(^^1^^,^^14^^,^^33^^)^. It should be borne in mind that some schwannomas and paragangliomas can be completely cystic and should therefore be included in the differential diagnosis^(^^33^^,^^34^^)^.

**Key point:** The management of retroperitoneal cystic lesions is a clinical dilemma, the main options being percutaneous biopsy, clinical follow-up, and even (in symptomatic cases) surgical excision^(^^33^^)^.

### Vascularization

Tumor vascularization facilitates the characterization of retroperitoneal masses. Among the hypervascular lesions, we highlight the paragangliomas. Moderately hypervascular tumors include leiomyosarcomas, myxofibrosarcoma, and other sarcomas. In contrast, hypovascular tumors comprise low-grade liposarcomas, lymphomas, and most of the benign tumors^(^^1^^)^.

### Lesions with a mantle growth pattern

The behavior of a lesion, especially its growth pattern, extent, and relationship with the adjacent structures, facilitates the differential diagnosis. The term mantle growth pattern applies to lesions involving adjacent structures with no obvious signs of infiltration.

**Lymphoma** - Lymphoma is the most common malignant retroperitoneal neoplasm, as well as being the most common small round cell tumor, and typically presents as a para-aortic or pelvic mass, involving the adjacent structures, with a homogeneous and hypovascular aspect^(^^1^^,^^3^^-^^5^^)^. Necrosis and calcifications are uncommon prior to treatment^(^^3^^)^. The standard examination for the diagnosis, staging, and in-treatment evaluation of lymphoma is ^18^F-FDG PET/CT. However, FDG avidity depends on factors such as the histological characteristics, grade, and proliferation of the tumor^(^^45^^,^^46^^)^. Some studies have shown that low-grade (indolent) lymphomas have less FDG avidity than do high-grade (aggressive) lymphomas, such as large B-cell lymphoma and nodular sclerosing Hodgkin lymphoma^(^^45^^,^^47^^)^. The treatment consists of chemotherapy combined with radiotherapy or immunotherapy, depending on the histological subtype^(^^46^^)^. One main differential diagnosis is metastatic lymph node enlargement, which has a similar appearance on imaging studies^(^^4^^,^^5^^)^.

**Figure 8:**
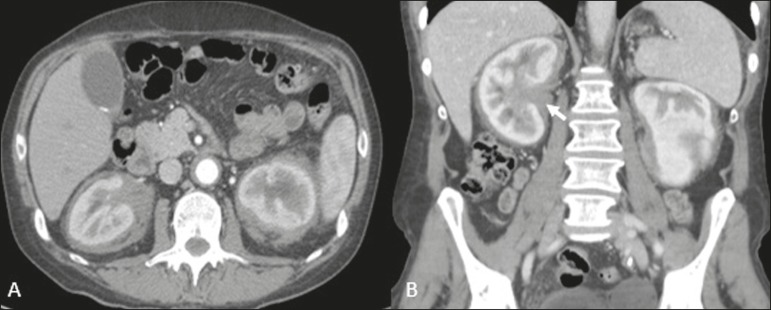
Mantle zone growth pattern. Erdheim-Chester disease in a 62-year-old male. CT scans (**A,B**) showing perirenal solid tissue (mantle zone growth pattern) surrounding the renal hilum (arrow in **B**). No bilateral hydronephrosis was observed.

**Erdheim-Chester disease** - A rare non-Langerhans cell histiocytosis of unknown origin, Erdheim-Chester disease is characterized by multiorgan inflammation composed of histiocytes and commonly affects males between 40 and 60 years of age. In 29% of the cases, there is retroperitoneal involvement, characterized by homogeneous soft-tissue attenuation on CT, as well as by a hypointense to isointense signal in T1-weighted MRI sequences and a hyperintense signal in T2-weighted MRI sequences^(^^3^^,^^5^^,^^48^^,^^49^^)^. Circumferential involvement of the aorta and the bilateral symmetrical perirenal space is characteristic of the disease. As shown in Figure 8, the ureters can also be involved^(^^3^^,^^48^^,^^49^^)^. The diagnosis is based on the identification of specific histopathological findings in an appropriate clinical and radiological context. Bone changes are common; biopsy is usually necessary in order to confirm the diagnosis, even in cases in which there is a high level of clinical suspicion^(^^48^^)^. Due to the rarity of the disease and the scarcity of randomized studies of the topic, there is little knowledge about the specific treatment, which is based on the use of corticosteroids, immunomodulatory agents, and cytotoxic agents, as well as other drugs^(^^48^^,^^49^^)^.

**Retroperitoneal fibrosis** - Retroperitoneal fibrosis is a rare condition that is more common in males and is classified as idiopathic or secondary^(^^3^^,^^4^^,^^50^^)^. The idiopathic form accounts for two thirds of all cases. The secondary form is related to neoplasms, infections, trauma, and other conditions. On imaging examinations, retroperitoneal fibrosis is characterized by homogeneous attenuation of the soft tissue surrounding the abdominal aorta and iliac arteries, with or without involvement of the adjacent structures, such as the ureters and the inferior vena cava, which would result in ureteral obstruction and venous thrombosis, respectively^(^^4^^,^^50^^)^. On MRI, it shows an intense signal in T2-weighted sequences and a enhancement pattern that varies according to the level of disease activity^(^^3^^)^. Treatment of the idiopathic form consists of corticosteroid therapy, and treatment with tamoxifen has been quite effective in some cases. In cases of secondary retroperitoneal fibrosis, the treatment is based on management of the underlying disease^(^^3^^,^^4^^,^^50^^)^.

**Key point:** Retroperitoneal lesions with a mantle growth pattern can be benign or malignant (e.g., lymphoma) and are typically treated with medications or chemotherapy, suggesting that percutaneous biopsy is an important tool in their initial clinical management.

## DIAGNOSTIC ALGORITHM AND CLINICAL MANAGEMENT

**Figure 9:**
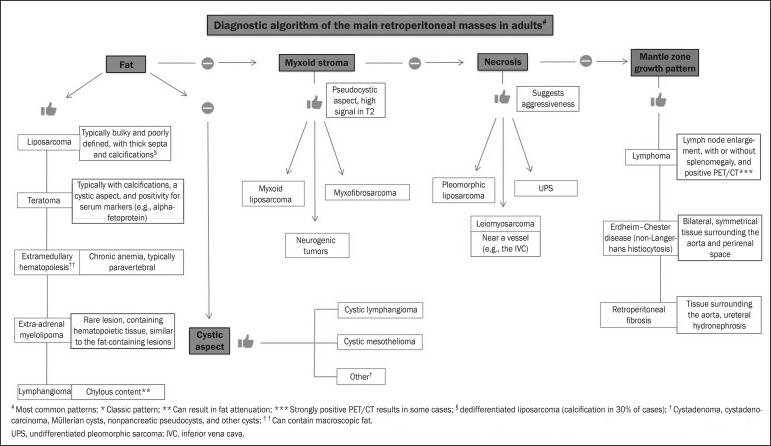
Algorithm demonstrating the practical evaluation of retroperitoneal masses based on the main image characteristics (fat, cystic aspect, myxoid stroma, necrosis, and a mantle zone growth pattern).

**Figure 10:**
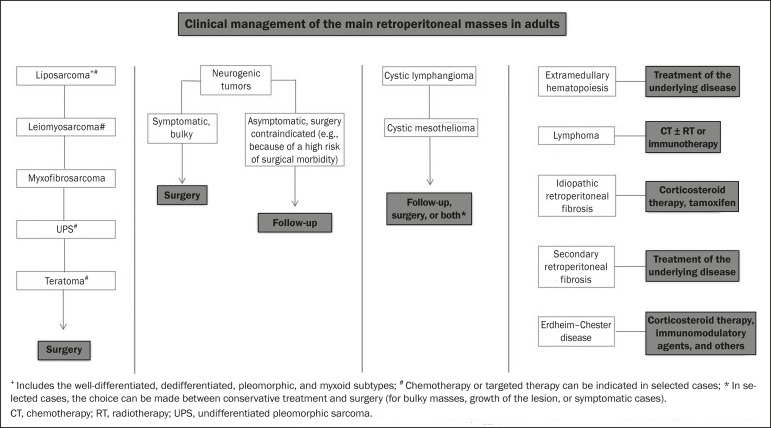
Clinical management of the main retroperitoneal masses.

We present an algorithm with the objective of facilitating the differential diagnosis of the main primary retroperitoneal masses by analyzing the imaging characteristics previously mentioned (Figure 9). Figure 10 deals with the main practices in the management of primary retroperitoneal lesions.

## CONCLUSION

Primary retroperitoneal lesions are rare and constitute a diagnostic challenge due to overlapping imaging findings. Knowledge of imaging characteristics helps narrow the differential diagnosis and guide clinical decision making.
